# Fructose‐1,6‐bisphosphate reverses hypotensive effect caused by L‐kynurenine in *Wistar* male rats

**DOI:** 10.14814/phy2.70033

**Published:** 2024-10-13

**Authors:** Anderson Velasque Catarina, Gisele Branchini, Rafael Andrade Caceres, Renata Streck Fernandes, Bruna Pasqualotto Costa, Kleiton Lima De Godoy Machado, Tiago Becker, Luis Fernando Ferreira, Katya Rigatto, Jarbas Rodrigues de Oliveira, Fernanda Bordignon Nunes

**Affiliations:** ^1^ Graduate Program in Pathology—Laboratory of Computational Molecular, and Cellular Biophysics—Universidade Federal de Ciências da Saúde de Porto Alegre (UFCSPA) Porto Alegre Brazil; ^2^ Department of Pharmacosciences Universidade Federal de Ciências da Saúde de Porto Alegre (UFCSPA) Porto Alegre Brazil; ^3^ Graduate Program in Health Sciences—Laboratory of Translational Physiology—Universidade Federal de Ciências da Saúde de Porto Alegre (UFCSPA) Porto Alegre Brazil; ^4^ Laboratory of Inflammation and Cellular Biophysics—Pontifícia Universidade Católica Do Rio Grande Do Sul (PUCRS) Porto Alegre Brazil; ^5^ Department of Plant Biology Universidade Federal de Viçosa (UFV) Viçosa Brazil; ^6^ Department of Mechanical Engineering Universidade Federal do Rio Grande do Sul Porto Alegre Brazil; ^7^ School of Electronics, Electrical Engineering and Computer Sciences Queen's University of Belfast Belfast UK; ^8^ Graduate Program in Medicine: Hepatology—Universidade Federal de Ciências da Saúde de Porto Alegre (UFCSPA) Porto Alegre Brazil

**Keywords:** fructose‐1,6‐bisphosphate, hypotension, inflammation, L‐kynurenine, potassium‐voltage‐gated channels

## Abstract

Hypotension is one of the main characteristics of the systemic inflammation, basically caused by endothelial dysfunction. Studies have shown that the amino acid L‐kynurenine (KYN) causes vasodilation in mammals, leading to hypotensive shock. In hypotensive shock, when activated by the KYN, the voltage‐gated potassium channel encoded by the family *KCNQ* (Kv7) gene can cause vasodilation. Fructose‐1,6‐bisphosphate (FBP) it is being considered in studies an anti‐inflammatory, antioxidant, immunomodulator, and a modulator of some ion channels (Ca2+, Na+, and K+). We analyzed the effects of KYN and FBP on mean blood pressure (MBP), systolic and diastolic (DBP) blood pressure, and heart rate variability (HRV) in *Wistar* rats. Results demonstrated that the administration of KYN significant decreased MBP, DBP, and increased HRV. Importantly, the FBP treatment reversed the KYN effects on MBP, DBP, and HRV. Molecular Docking Simulations suggested that KYN and FBP present a very close estimated free energy of binding and the same position into structure of *KCNQ4*. Our results did demonstrate that FBP blunted the decrease in BP, provoked by KYN. Results raise new hypotheses for future and studies in the treatment of hypotension resulting from inflammation.

## 1 INTRODUCTION

Hypotension is one of the main characteristics of systemic inflammatory response syndrome, basically caused by endothelial dysfunction (Boisrame‐Helms et al., 2013; Dolmatova et al., 2021; Feihl et al., 2013; Kimmoun et al., 2013). Endothelial dysfunction causes a reduction in systemic vascular resistance and poor distribution of blood in the microcirculation, compromising tissue perfusion, and it is correlated with poor prognosis up to organ failure (Dolmatova et al., 2021; Feihl et al., 2013; Sakr et al., 2012; Yin et al., 2019).

During the inflammatory process, there is an increase in pro‐inflammatory cytokines, such as tumor necrosis factors (TNF‐α) and interferon‐γ (IFNγ), which induce the expression of the protein indoleamine 2,3‐dioxygenase (IDO), activating the kynurenine pathway (Fazio et al., 2017; Sakakibara et al., 2015; Y. Wang et al., 2010). Indoleamine 2,3‐dioxygenase transforms the essential amino acid tryptophan into KYN in the most varied of cell types (Fazio et al., 2017; Sakakibara et al., 2015; Y. Wang et al., 2010). Studies have shown that the amino acid KYN causes vasodilation in the arteries of mammals such as rats and humans, leading to hypotensive shock, especially correlated with inflammation (Fazio et al., 2017; Sakakibara et al., 2015; Y. Wang et al., 2010; Worton et al., 2021). IFNγ increases IDO protein expression in human aortic endothelial cells, leading to an increase in KYN levels to 10 μM in culture medium (Sakakibara et al., 2015; Q. Wang et al., 2013). These reports point out that inflammation plays an important role in increasing plasma levels of KYN.

Fructose‐1,6‐bisphosphate is a biphosphorylated sugar and a high‐energy intermediate metabolite of the glycolytic pathway (Catarina et al., 2018; Fructose 1,6‐Bisphosphate: A Summary of Its Cytoprotective Mechanism. Alva et al., 2016; Kirtley & Mckay, 1977; Chlouverakis, 1968). Studies have suggested that FBP is an anti‐inflammatory (Alva et al., 2016; Nunes et al., 2003), antioxidant (Alva et al., 2016; De Mello et al., 2011) and, also, that it acts as a modulator of some ion channels (Ca^2+^, Na^+^ e K^+^). FBP is also considered an inducer of the membrane potassium channels stabilizer, preventing the reduction of intracellular K^+^ concentration (Alva et al., 2016; de Fraga et al., 2011).

In physiological conditions, hemodynamic changes usually occur, leading to alterations in blood pressure (BP). Those changes are usually blunted by the autonomic nervous system that adapt the heart rate (HR) to maintain BP (Vanderlei et al., 2009). The agility in changing HR expresses the heart rate variability (HRV), which it is crucial to BP stabilization (Rajendra Acharya et al., 2007; Taccone et al., 2013; Vanderlei et al., 2009).

Considering the complexity of molecules design that selectively inhibit the KYN pathway, our goal was to demonstrate, by spectral analysis, whether FBP administration would improve the hemodynamic responses of Wistar rats treated with KYN. Furthermore, we investigate, in silico, a putative site of binding for FBP and KYN in a potassium channel that could be involved in those hemodynamic alterations.

## 2 MATERIALS AND METHODS

### 2.1 Animals

Male *Wistar* Rats (20–24 weeks old), weighting between 350 and 450 grams, from the bioterium of Universidade Federal de Ciências da Saúde de Porto Alegre (UFCSPA) were divided into three groups: (i) control group (Naive): they did not receive any type of intervention or drug administration; (ii) KYN group: the animals received 100 mg/Kg of L‐kynurenine (SIGMA‐ALDRICH) (Pocivavsek et al., 2017) intraperitoneally; and (iii) KYN + FBP group: the animals received 100 mg/Kg of L‐kynurenine and, at the same time, 500 mg/Kg of Fructose‐1,6‐bisphosphate (SIGMA‐ALDRICH) (Bordignon Nunes et al., 2002; Catarina et al., 2018; Nunes et al., 2003) both intraperitoneally.

In accordance with the Guiding Principles in the Care and Use of Animals approved by the Council of the American Physiological Society, the animals were kept in cages, in an air‐conditioned room, relative humidity ranging between 55 and 65%, a 12 h light–dark cycle, and a temperature of 22 ± 2°C, with free access to food and water. The rats were fed with food for laboratory animals (rats) NUVILAB CR‐1, supplied by QUIMTIA S.A. The experimental protocol was approved by the Ethics Committee on the Use of Animals (ECUA) of UFCSPA (protocol number 249/19).

### 2.2 Hemodynamic evaluation

One hour after all treatments, the animals were anesthetized with ketamine (80 mg/Kg) and xylazine (20 mg/Kg) intraperitoneally. Under anesthesia, a polyethylene catheter (PE‐50) was inserted into the right carotid to record arterial blood pressure (ABP) for 10 min (sample rate = 2000 Hz/channel). The analogical signals of systolic and diastolic blood pressure were digitalized by a data‐acquisition system (Windaq‐AT/CODAS, Dataq 143 Instruments Inc., OH, USA). The data were analyzed by spectral analysis to assess the sympathovagal balance in the cardiovascular system (Fernandes et al., 2021; Rigatto et al., 2013).

### 2.3 Autonomic evaluation

After detecting the pulse intervals, the heart period was automatically calculated on a beat‐to‐beat basis as the time interval between two consecutive systolic peaks or pulse interval (PI). All detections were carefully checked to avoid erroneous or missed beats. Sequences of 200–250 beats were randomly chosen (Dabiré et al., 1998; Dias da Silva et al., 2006) and if they presented non‐stationary episodes, they were discarded and a new random selection was performed. Stationarity of the series was tested as previously reported (Porta et al., 2004). Frequency domain analysis of HRV was performed with an autoregressive algorithm (Porta et al., 2004) on the PI interval sequences (tachogram). The power spectral density was calculated for each time series.

In this study, two spectral components were considered: low frequency (LF), from 0.25 to 0.75 Hz; and high frequency (HF), from 0.75 to 3.00 Hz (Dabiré et al., 1998; Dias da Silva et al., 2006) The spectral components (ms^2^) were expressed in absolute (a) and normalized (nu) units. Normalization consisted of dividing the power of a given spectral component by the total power, then multiplying the ratio by 100. In a reduced variability condition, linear methodologies have poor applicability (Montano et al., 1994). Thus, the non‐linear approach provides a new perspective in the investigation of neural control of the cardiovascular system (Casali et al., 2008; Porta et al., 2021).

### 2.4 Protein structure selection

The crystallographic structure of KCNQ4 receptor was retrieved from the Protein Data Bank (Berman et al., 2000; Burley et al., 2021). The atomic coordinate of the structure of KCNQ4 (PDB access code 7BYM) with resolution at 3.1 Å was used on the molecular docking simulations (Li et al., 2021).

### 2.5 Molecular docking simulation protocol

The molecular docking experiments were performed by AutoDock Vina (Manuscript, 2011) in PyRx 0.8v (Dallakyan & Olson, 2016) Autodock Vina employs a gradient‐based conformational search space by a grid box defined by the box center coordinates and its dimensions of x, y, and z in grid resolution internally assigned to 1 Å. For calculations of docking protocol performed in the docking simulations a resolution of 0.375 Å to the mesh affinity (grid), consisting of 13.7674 × 9.7760 × 12.5092 (x, y, z) angstrom and center at 186.1500 × 181.8968 × 157.7945 (xyz‐coordinates) points adjusted at the retigabine binding site. The exhaustivness were set to 20 to control how many times the calculations are repeated. The scoring of the generated docking poses and ranking of the ligands were based on the Vina empirical scoring function.

### 2.6 Statistical analysis

Using GraphPad Prism 5.0 (GraphPad Software, San Diego, CA), the One‐way analysis of variance (ANOVA) followed by Tukey's multiple comparison post‐test was used for comparisons between groups in the assays. All results are presented as mean ± standard deviation (SD) of values of each group. A *p* value ≤ 0.05 was considered statistically significant.

## 3 RESULTS

### 3.1 Mean, diastolic, and systolic blood pressure

**FIGURE 1:**
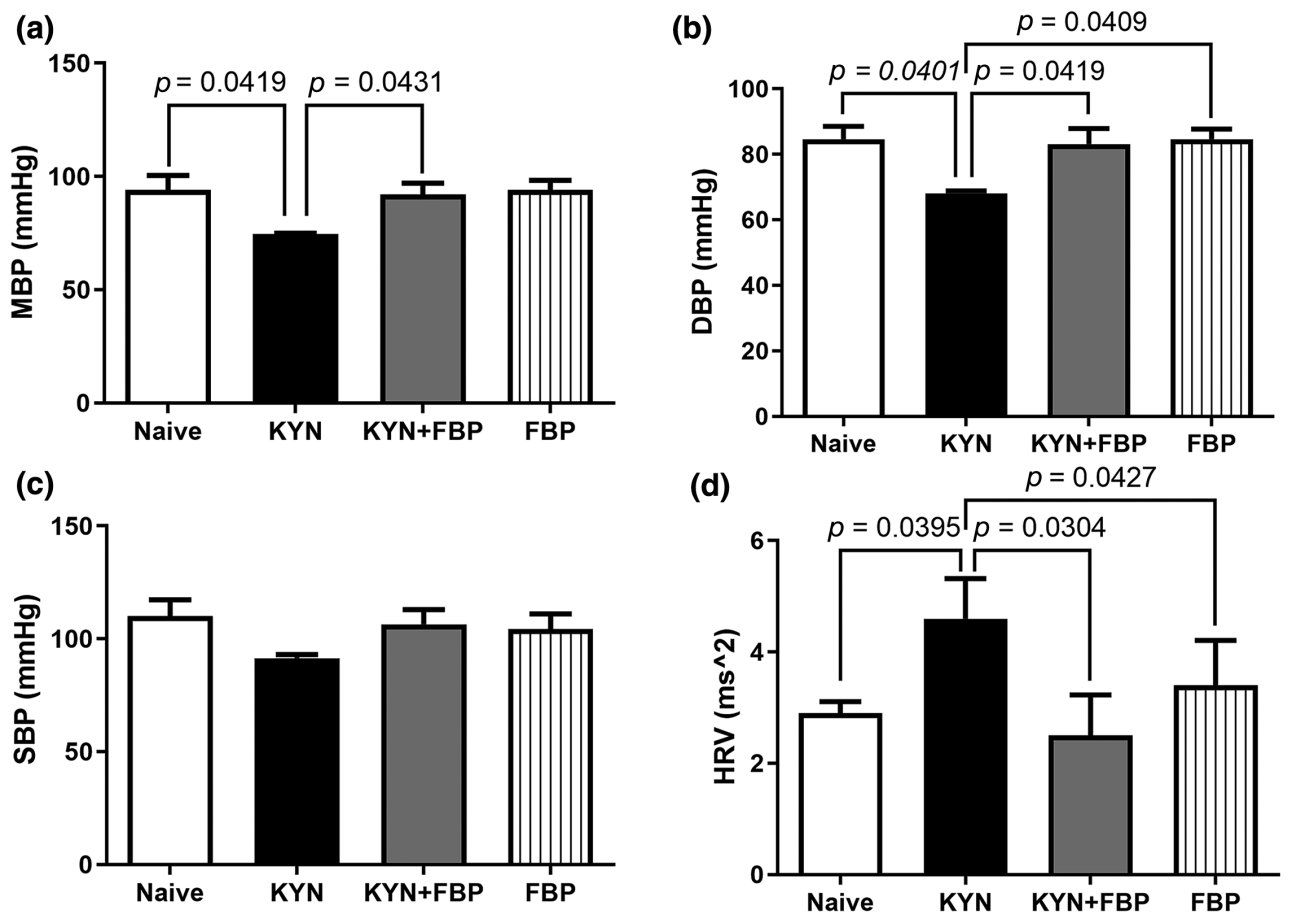
Blood pressure and heart rate measures in response to KYN, KYN + FBP and FBP administrations. (a) Mean blood pressure (MBP), with a significant reduction in the KYN group compared to the naive and KYN + FBP groups. (b) Diastolic blood pressure (DBP), with a significant reduction in the KYN group compared to the other groups. (c) Systolic blood pressure (SBP), with no significant difference when comparing groups. (d) Heart rate variability (HRV), with significantly greater variation in the KYN group compared to the naïve and KYN + FBP groups. Naive and KYN group (*n* = 7); KYN + FBP (*n* = 6). Data are presented as mean ± standard deviation.

The administration of KYN significantly reduced the mean BP (MBP) and diastolic BP (DBP), compared to the values of the naïve and KYN + FBP groups (*p* < 0.05), while the values of MBP and DBP of the group treated with FBP were similar to the naïve group (Figure 1). There was no difference in systolic blood pressure among groups (Figure 1c).

### 3.2 Spectral analysis of HRV

The HRV (*p* < 0.0224) (Figure 1d), LFa (*p* < 0.0231) and HFa (*p* < 0.0185) were significantly higher in KYN compared to naïve and KYN + FBP groups. These results indicate that, in absolute values, both sympathetic and parasympathetic system were increased in KYN group, which provoked the increase in HRV. On the other hand, the normalized units (LFnu and HFnu), that reflect the sympathovagal balance, did not show a difference among the groups (Table 1).

**TABLE 1:** Spectral and symbolic analysis results. Data represent medians and minimum and maximum values.

	CO	KYN	KYN + FPB	FBP	*p*
Spectral analysis					
HRV (ms^2^)	2.75 (1.81–3.7)	6.25 (3.19–9.31)^a^	2.93 (1.32–4.54)	2.93 (1.91–3.82)	0.03
LFa (ms^2^)	0.98 (0.61–1.36)	2.52 (0.73–4.32)^a^	1.06 (0.39–1.73)	1.02 (0.93–1.13)	0.05
HFa (ms^2^)	1.40 (1.17–1.64)	3.38 (1.52–5.24)^a^	1.22 (0.61–1.83)	1.48 (1.22–1.74)	0.01
LFnu	0.42 (0.33–0.51)	0.41 (0.29–0.53)	0.32 (0.19–0.45)	0.43 (0.35–0.56)	0.16
HFnu	0.58 (0.49–0.67)	0.46 (0.28–0.64)	1.22 (0.55–1.89)	0.59 (0.50–0.69)	0.058
LF/HFratio	0.75 (0.62–0.88)	0.96 (0.41–1,51)	0.69 (0.21–1.18)	0.78 (0.69–0.93)	0.11
Symbolic analysis (%)					
0 V pattern	0.012 (0.6–0.14)	0.14 (0.8–0.32)	0.13 (0.6–0.20)	0.014 (0.7–0.16)	0.14
1 V pattern	0.38 (0.29–0,47)	0.33 (0.16–0.44)	0.42 (4.39–1.49)	0.40 (0.31–0,49)	0.1
2LV pattern	14 (04–0.18)	0.10 (0.4–0.17)	0.12 (0.7–0.20)	15 (05–0.20)	0.14
2UV pattern	0.36 (0.28–0.51)	28 (0.13–0.38)	0.33 (0.13–0.36)	0.38 (0.29–0.53)	0.13

*Note*: Naïve = control animals, (*n* = 7); KYN = animals treated with L‐kynurenine, (*n* = 7); KYN + FBP = animals treated with L‐kynurenine+Fructose‐1,6‐bisphosphate, (*n* = 6).

Abbreviations: a, absolute; HF, Hight frequency component; HRV, Heart rate variability; LF, Low frequency component; nu, normalized.

^a^ Medians from KYN group are significantly different of the medians from naive and KYN + FBP groups (*p* < 0.05; One‐way analysis of variance (ANOVA) followed by Tukey's multiple comparison post‐test).

### 3.3 Molecular docking simulations of Fructose‐1,6‐bisphosphate (FBP) and L‐kynurenine (KYN)

**FIGURE 2:**
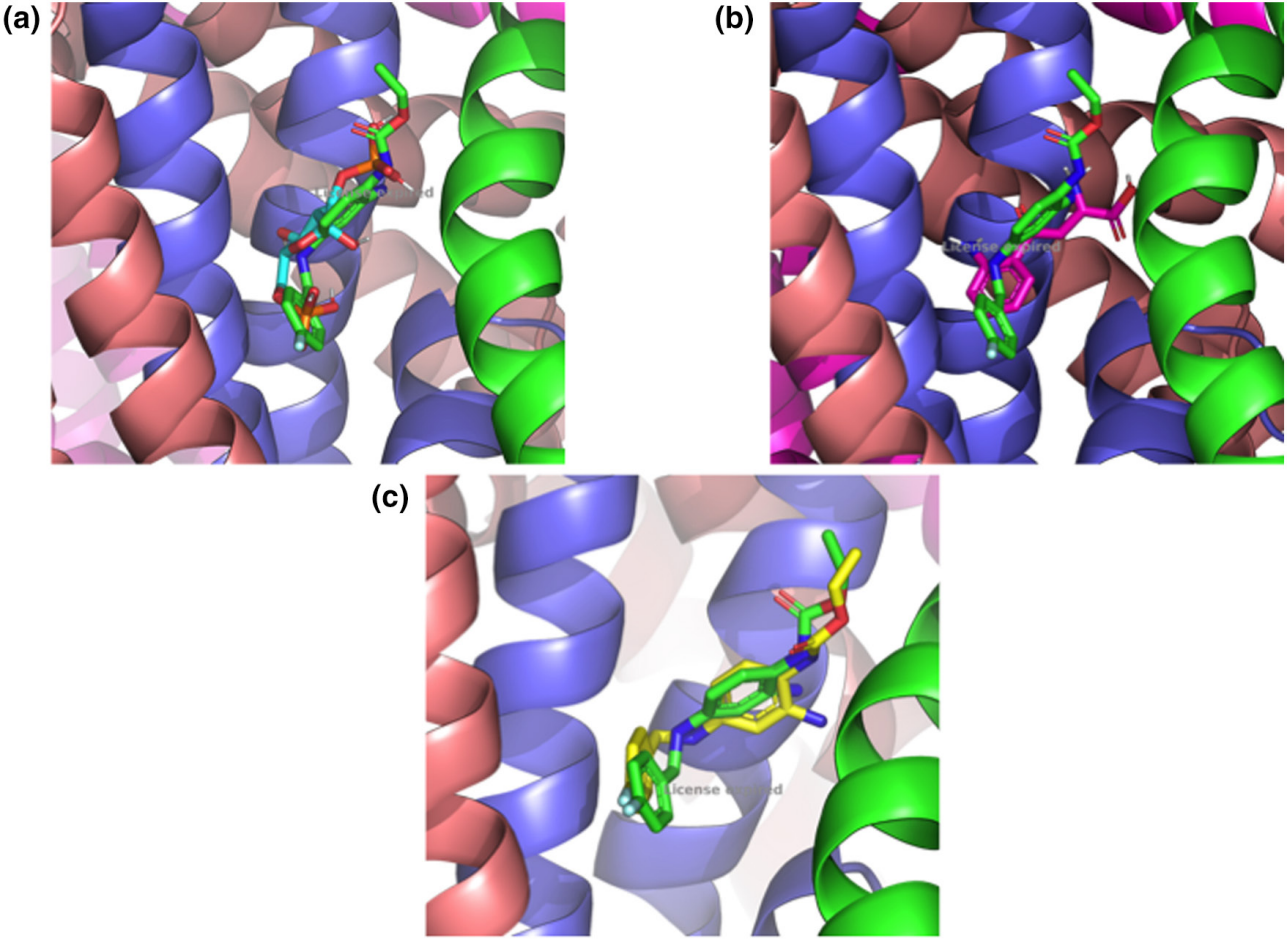
Docking validation structures. *KCNQ4* voltage‐gated potassium channel crystallographic structure is represented as cartoon. (a) The crystalographic structure of retigabine (green) and FBP docking pose (cyan), (b) The crystalographic structure of retigabine (green) and KYN docking pose (pink), and (c) The crystalographic structure of retigabine (green) and retigabine docking pose (yellow) are represented in stick model. Elaborated using Pymol 2.3.3.

**FIGURE 3:**
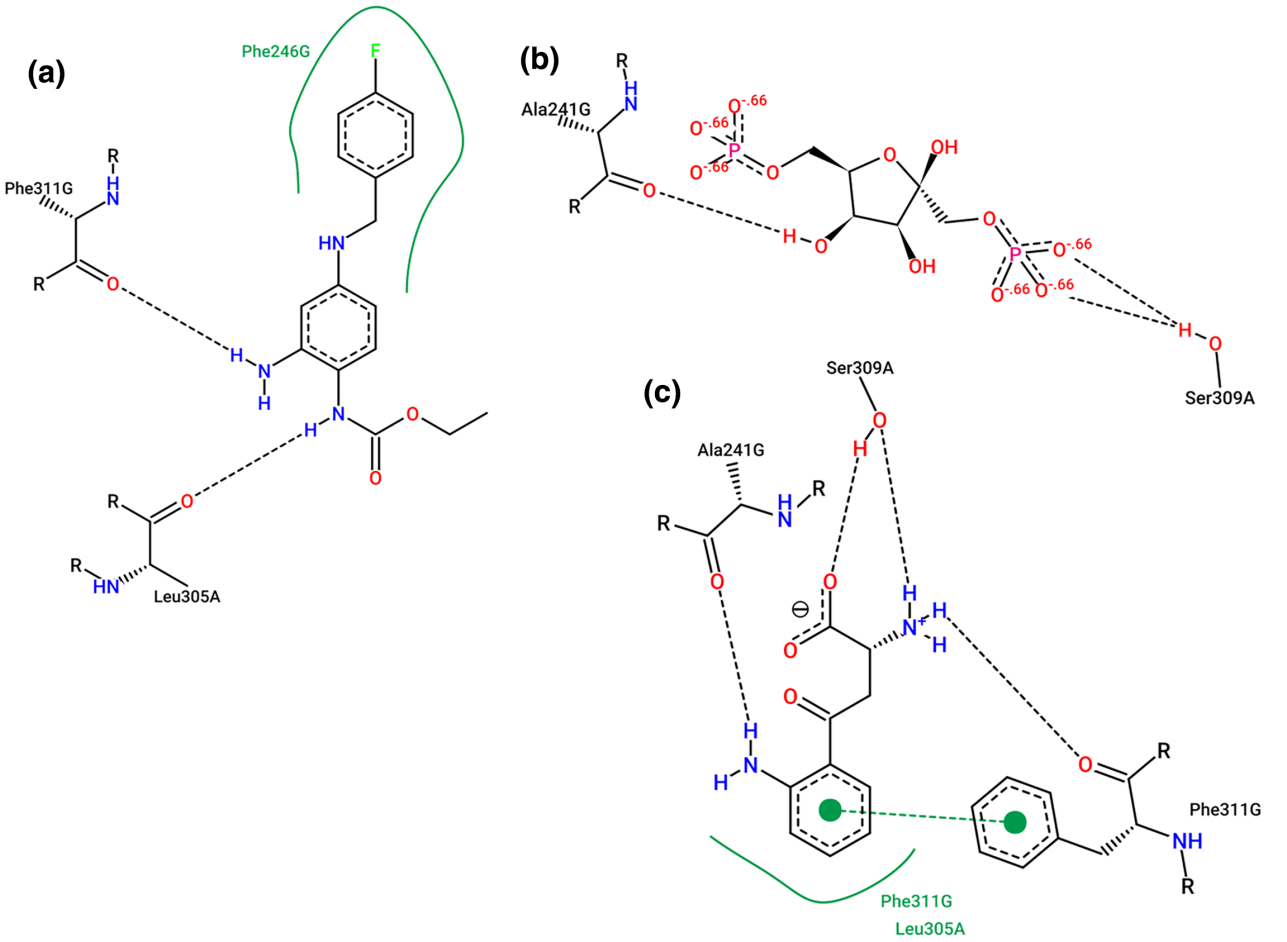
Two‐dimensional diagrams of complexes of interaction with *KCNQ4* voltage‐gated potassium channel. Directed bonds between protein (channel) and ligand are drawn as dashed lines and the interacting protein residues and the ligand are visualized as structure diagrams. Hydrophobic contacts are represented more indirectly through spline sections highlighting the hydrophobic parts of the ligand and the label of the contacting residue. (a) *KCNQ4* voltage‐gated potassium channel in liganding interaction with retigabine. (b) *KCNQ4* voltage‐gated potassium channel in liganding interaction with FBP. (c) *KCNQ4* voltage‐gated potassium channel in liganding interaction with KYN. Figure generated using PoseView.

The docking methodology was used to identify the possible mode of association of FBP and KYN on the KCNQ4 voltage‐gated potassium channel using the retigabine biding site as reference. The best binding conformation of FBP (PubChem CID 10267), which has better estimated free energy of binding of −5,60 Kcal/mol, (Figure 2a) and the best binding conformation of KYN (PubChem CID 161166) furnish an estimated free energy of binding of −6,7 Kcal/mol (Figure 2b) were selected. The same protocol described previously was applied to validate the recovery of retigabine position (−7,7 Kcal/mol) on the crystallographic structure. It is important to note that both complexes present a very close estimated free energy of binding and the same position into structure of KCNQ4 shown in Figure 2c, suggesting that FBP and KYN could be a channel competitor of retigabine. The KCNQ4 voltage‐gated potassium channel and ligands interactions are shown on Figure 3.

## 4 DISCUSSION

Patients those affected by systemic inflammation require constant monitoring of hemodynamic conditions, and, in the event of hypotension, volume replacement measures and the use of vasopressors are taken. It is essential to understand the origin or mechanisms involved in vasodilation, in order to find therapeutic strategies that can be used in the course of the disease to prevent hypotension and improve the prognosis of these patients (Boisrame‐Helms et al., 2013;Dolmatova et al., 2021; Feihl et al., 2013; Kimmoun et al., 2013). In this way, we evaluated effect of FBP in parameters related to hemodynamic changes. The main finding of our study was that FBP administration, concomitantly with KYN administration, prevented the hypotension triggered by the amino acid KYN and reduced the heart rate variability. The administration of KYN caused a reduction in the DBP, MBP, and increased the HRV (Figure 1). All those alterations were significantly attenuated by the FBP treatment (Figure 1), indicating that FBP was effective against the main effect of inflammatory response syndrome (Dolmatova et al., 2021; Feihl et al., 2013; Kimmoun et al., 2013).

Hemodynamic changes in blood pressure are usually attenuated by the autonomic nervous system through heart rate (HR) adaptation (Vanderlei et al., 2009). Although there was no difference in sympathovagal balance among groups, evidenced by the LF/HF ratio similar among the experimental groups (Table 1), the heart rate variability was higher in KYN group due to the increase in total power variability, FBP treatment reversed the KYN effects on HRV (Figure 1d and Table 1). According to the literature, HRV is defined as the heart rate ability to blunt the variation in arterial BP, either in physiological or pathophysiological situations (Dabiré et al., 1998; Rigatto et al., 2013; Vanderlei et al., 2009), directly proportional to the LF and HF magnitude and can be used to for monitor the patient's evolution (Moss et al., 2018; Neto et al., 2004; Sassi et al., 2015). The fact of that FBP administration was able to reduce HRV suggests a reduction in the requirement of the autonomic system to attenuate the reduction of blood pressure, evidencing a possible protective effect of FBP in the cardiovascular system during hypotension caused by KYN.

The autonomic balance, evidenced by the LF/HF ratio, was similar among the experimental groups (Table 1), probably due to the similarity in LFnu and HFnu, demonstrating a proportional participation of sympathetic and parasympathetic nervous system, respectively. Thus, the final effect over the heart is the increase in time between heart beats, exactly what spectral analysis measures to identify LFa and HFa. Whether the FBP is competitively binding to the same receptor of KYN or inducing a bypass in the intracellular route remains to be determined, but our results demonstrated that FBP can attenuate the decrease in BP without any harm to the studied parameters. Regarding to the autonomic modulation, the LFa, or sympathetic modulation in absolute values, was increased in KYN group probably in response to the decrease in BP.

In silico analysis are useful to identify possible interactions between virtually any molecules, through the blind molecular docking simulations methodology, which predicts the orientations of possible modes of association of one molecule when it binds to the other (receptor‐ligand), through the AutoDock Vina (Ng et al., 2011) em PyRx 0,8v (Dallakyan & Olson, 2016). The best binding conformation of FBP, which has better estimated free energy of binding of −5,60 Kcal/mol, (Figure 2a) and the best binding conformation of KYN furnish an estimated free energy of binding of −6,7 Kcal/mol (Figure 2b) were selected. Molecular docking simulations were performed not only to confirm the literature on the association of KYN with Kv7.4 channel (Sakakibara et al., 2015; Wolowczuk et al., 2021; Worton et al., 2021), but mainly to obtain indications of possible interactions of the FBP with the same channel. It is important to note that both complexes present a very close estimated free energy of binding and the same position into structure of *KCNQ4* shown in Figure 2c, suggesting that FBP and KYN could be a channel competitor of Retigabine, with possible and interesting interactions (Figure 3).

The Kv7.4 channel, as well as other components of KCNQ family, is present in the vascular smooth muscle of humans and rodents and induces vasodilation during the inflammatory process (Kamouchi et al., 1999; Kimmoun et al., 2013; Sakakibara et al., 2015). The metabolite KYN acts as an activator of the potassium voltage‐gated channels Kv7 (Sakakibara et al., 2015), and has been demonstrated in rats and humans that, during sepsis, it could potentially contribute to hypotension during the inflammatory process (Tattevin et al., 2010; Y. Wang et al., 2010; Changsirivathanathamrong et al., 2011; Stone & Darlington, 2002; Stone et al., 2013; Wolowczuk et al., 2021). During the inflammatory process, there is an increase in pro‐inflammatory cytokines, such as TNF‐α and IFNγ, which induce the expression of the protein IDO, thus activating the KYN pathway. Indoleamine 2,3‐dioxygenase transforms the essential amino acid tryptophan into KYN in the most varied cell types, including vascular endothelial cells as the main site of this pathway (Kamouchi et al., 1999; Kimmoun et al., 2013; Sakakibara et al., 2015). According to Kimmoun et al. (Kimmoun et al., 2013), the behavior of vascular smooth muscle cells (VSMCs) is controlled by the voltage‐sensitive calcium channels, depending on the potassium channels, which control the cell membrane polarization of endothelial cells and VSMCs. In this way, once KYN causes vasodilation via potassium channels, and considering the possible molecular interaction between FBP and Kv7.4 (indicated by molecular docking), it is possible to suggest that FBP can competitively bind to the same receptor of KYN, given the FBP the ability to reverse the decrease in BP, provoked by KYN. Evidence from the literature also indicate that FBP acting as a modulator of some ion channels (Ca^2+^, Na^+^ e K^+^), being an inducer of the stabilization of potassium channels in the membrane (Alva et al., 2016; Roig et al., 1997). Nonetheless, if FBP is a competitor of the same binding site or it is inducing a bypass in an intracellular route remains to be elucidated.

## 5 CONCLUSION

Once the hypotension is one of the main characteristics of systemic inflammatory response syndrome, basically caused by generalized vasodilatation, the possibility of the use of FBP in prevent this hemodynamic alteration can spread the possibility of treatments and therapeutically approaches. Results showed by the present study, those results provide evidence for future studies and, also, to for design new therapeutically approaches to the treatment of hypotensive shock in inflammation.

## AUTHOR CONTRIBUTIONS

Catarina AV contributed to the project development, data collection, data analysis, and manuscript writing. Caceres RA and Rigatto KV contributed to project development, data collection, data analysis, and manuscript writing. Costa BP and Machado KL contributed to data analysis, manuscript writing/editing. Branchini G and Nunes FB contributed to study design, project development, data analysis, and manuscript writing/editing.

## CONFLICT OF INTEREST STATEMENT

The authors declare that the study did not obtain, nor did it receive, sources of funding for this work, nor does it have funding contracts with any institution for the preparation of this paper.

## ETHICS STATEMENT

The experimental protocol was approved by the Ethics Committee on the Use of Animals (ECUA) of UFCSPA (protocol number 249/19).

## Supporting information


Data S1.

## References

[phy270033-bib-0001] Alva, N. , Alva, R. , & Carbonell, T. (2016). Fructose 1,6‐bisphosphate: A summary of its cytoprotective mechanism. Current Medicinal Chemistry, 23(39), 4396–4417. 10.2174/0929867323666161014144250 27758716

[phy270033-bib-0003] Berman, H. M. , Battistuz, T. , Bhat, T. N. , Bluhm, W. F. , Bourne, P. E. , Burkhardt, K. , Feng, Z. , Gilliland, G. L. , Iype, L. , Jain, S. , Fagan, P. , Marvin, J. , Padilla, D. , Ravichandran, V. , Schneider, B. , Thanki, N. , Weissig, H. , Westbrook, J. D. , & Zardecki, C. (2000). The Protein Data Bank. Acta Crystallographica Section D: Biological Crystallography, 28(1), 235–242. 10.1107/s0907444902003451 12037327

[phy270033-bib-0004] Boisrame‐Helms, J. , Kremer, H. , Schini‐Kerth, V. , & Meziani, F. (2013). Endothelial dysfunction in sepsis. Current Vascular Pharmacology, 11(2), 150–160. 10.2174/157016113805290317 23506494

[phy270033-bib-0005] Bordignon Nunes, F. , Simões Pires, M. G. , Alves Filho, J. C. F. , Wächter, P. H. , & De Oliveira, J. R. (2002). Physiopathological studies in septic rats and the use of fructose 1,6‐bisphosphate as cellular protection. Critical Care Medicine, 30(9), 2069–2074. 10.1097/00003246-200209000-00020 12352043

[phy270033-bib-0006] Burley, S. K. , Bhikadiya, C. , Bi, C. , Bittrich, S. , Chen, L. , Crichlow, G. V. , Christie, C. H. , Dalenberg, K. , Di Costanzo, L. , Duarte, J. M. , Dutta, S. , Feng, Z. , Ganesan, S. , Goodsell, D. S. , Ghosh, S. , Green, R. K. , Guranović, V. , Guzenko, D. , Hudson, B. P. , … Zhuravleva, M. (2021). RCSB Protein Data Bank: powerful new tools for exploring 3D structures of biological macromolecules for basic and applied research and education in fundamental biology, biomedicine, biotechnology, bioengineering and energy sciences. Nucleic Acids Research, 49, 437–451. 10.1093/nar/gkaa1038 PMC7779003 33211854

[phy270033-bib-0007] Casali, K. R. , Casali, A. G. , Montano, N. , Irigoyen, M. C. , Macagnan, F. , Guzzetti, S. , & Porta, A. (2008). Multiple testing strategy for the detection of temporal irreversibility in stationary time series. Physical Review E—Statistical, Nonlinear, and Soft Matter Physics, 77(6 Pt 2), 066204. 10.1103/PhysRevE.77.066204 18643347

[phy270033-bib-0008] Catarina, A. V. , Luft, C. , Greggio, S. , Venturin, G. T. , Ferreira, F. , Marques, E. P. , Rodrigues, L. , Wartchow, K. , Leite, M. C. , Gonçalves, C. A. , Wyse, A. T. S. , Da Costa, J. C. , De Oliveira, J. R. , Branchini, G. , & Nunes, F. B. (2018). Fructose‐1,6‐bisphosphate preserves glucose metabolism integrity and reduces reactive oxygen species in the brain during experimental sepsis. Brain Research, 1698, 54–61. 10.1016/j.brainres.2018.06.024 29932894

[phy270033-bib-0009] Changsirivathanathamrong, D. , Wang, Y. , Rajbhandari, D. , Maghzal, G. J. , Mak, W. M. , Woolfe, C. , Anzca, F. F. , Duflou, J. , Gebski, V. , Remedios, C. G. , Celermajer, D. S. , & Stocker, R. (2011). Tryptophan metabolism to kynurenine is a potential novel contributor to hypotension in human sepsis. Critical Care Medicine, 39(12), 2678–2683. 10.1097/CCM.0b013e31822827f2 21765346

[phy270033-bib-0010] Chlouverakis, C. (1968). The lipolytic action of fructose‐1‐6‐diphosphate. Metabolism, 17(8), 708–716. 10.1016/0026-0495(68)90055-3 5676217

[phy270033-bib-0011] Dabiré, H. , Mestivier, D. , Jarnet, J. , Safar, M. E. , & Chau, N. P. (1998). Quantification of sympathetic and parasympathetic tones by nonlinear indexes in normotensive rats. The American Journal of Physiology, 275(4), H1290–H1297. 10.1152/ajpheart.1998.275.4.H1290 9746478

[phy270033-bib-0012] Dallakyan, S. , & Olson, A. J. (2016). Small‐molecule library screening by docking with PyRx. Chemical Biology: Methods and Protocols, 1263, 243–250. 10.1007/978-1-4939-2269-7 25618350

[phy270033-bib-0013] de Fraga, R. S. , Heinen, P. E. T. , Kruel, C. R. P. , Molin, S. D. , Mota, S. M. , Cerski, C. T. S. , Gasperin, G. , Souto, A. A. , de Oliveira, J. R. , & Alvares‐da‐Silva, M. R. (2011). Fructose 1‐6 bisphosphate versus University of Wisconsin solution for rat liver preservation: Does FBP prevent early mitochondrial injury? Transplantation Proceedings, 43(5), 1468–1473. 10.1016/j.transproceed.2011.02.023 21693219

[phy270033-bib-0014] De Mello, R. O. , Lunardelli, A. , Caberlon, E. , Machado, C. , De Moraes, B. , Christ, R. , da Costa, V. L. , da Silva, G. V. , da Silva Scherer, P. , Buaes, L. E. , da Silva Melo, D. A. , Donadio, M. V. , Nunes, F. B. , & de Oliveira, J. R. (2011). Effect of N‐acetylcysteine and fructose‐1,6‐bisphosphate in the treatment of experimental sepsis. Inflammation, 34(6), 539–550. 10.1007/s10753-010-9261-9 20882329

[phy270033-bib-0015] Dias da Silva, V. J. , Montano, N. , Salgado, H. C. , & Fazan Júnior, R. (2006). Effects of long‐term angiotensin converting enzyme inhibition on cardiovascular variability in aging rats. Autonomic Neuroscience, 124, 49–55. 10.1016/j.autneu.2005.11.004 16439186

[phy270033-bib-0016] Dolmatova, E. V. , Griendling, K. K. , Wang, K. , & Mandavilli, R. (2021). The effects of sepsis on endothelium and clinical implications. Cardiovascular Research, 117, 60–73. 10.1093/cvr/cvaa070 32215570 PMC7810126

[phy270033-bib-0017] Fazio, F. , Carrizzo, A. , Lionetto, L. , Damato, A. , Capocci, L. , Simmaco, M. , Nicoletti, F. , & Vecchione, C. (2017). Vasorelaxing action of the kynurenine metabolite, xanthurenic acid: The missing link in endotoxin‐induced hypotension? Frontiers in Pharmacology, 8, 214. 10.3389/fphar.2017.00214 28507519 PMC5410560

[phy270033-bib-0018] Feihl, F. , Waeber, B. , & Liaudet, L. (2013). The hemodynamics of septic shock: A historical perspective. Current Vascular Pharmacology, 11(2), 133–138. 10.2174/157016113805290173 23506492

[phy270033-bib-0019] Fernandes, R. S. , Dias, H. B. , Amaral, W. , Jaques, D. S. , Becker, T. , & Rigatto, K. (2021). Assessment of alamandine in pulmonary fibrosis and respiratory mechanics in rodents. Journal of the Renin‐Angiotensin‐Aldosterone System, 2021, 9975315. 10.1155/2021/9975315 34285714 PMC8265028

[phy270033-bib-0022] Kamouchi, M. , Droogmans, G. , & Nilius, B. (1999). Membrane potential as a modulator of the free intracellular Ca2+ concentration in agonist‐activated endothelial cells. General Physiology and Biophysics, 18(2), 199–208. 10517293

[phy270033-bib-0023] Kimmoun, A. , Ducrocq, N. , & Levy, B. (2013). Mechanisms of vascular hyporesponsiveness in septic shock. Current Vascular Pharmacology, 11, 139–149. 10.2174/157016113805290245 23506493

[phy270033-bib-0024] Kirtley, M. E. , & Mckay, M. (1977). Fructose‐1,6‐bisphosphate, a regulator of metabolism. Molecular and Cellular Biochemistry, 18(2–3), 141–149. 10.1007/BF00280279 342914

[phy270033-bib-0025] Li, T. , Wu, K. , Yue, Z. , Wang, Y. , Zhang, F. , & Shen, H. (2021). Structural basis for the modulation of human KCNQ4 by small‐molecule drugs. Molecular Cell, 81(1), 25–37. 10.1016/j.molcel.2020.10.037 33238160

[phy270033-bib-0026] Montano, N. , Ruscone, T. G. , Porta, A. , Lombardi, F. , Pagani, M. , & Malliani, A. (1994). Power spectrum analysis of heart rate variability to assess the changes in sympathovagal balance during graded orthostatic tilt. Circulation, 90(4), 1826–1831. 7923668 10.1161/01.cir.90.4.1826

[phy270033-bib-0027] Moss, R. , Sachse, F. B. , Moreno‐Galindo, E. G. , Navarro‐Polanco, R. A. , Tristani‐Firouzi, M. , & Seemann, G. (2018). Modeling effects of voltage dependent properties of the cardiac muscarinic receptor on human sinus node function. PLoS Computational Biology, 14(10), e1006438. 10.1371/journal.pcbi.1006438 30303952 PMC6197694

[phy270033-bib-0028] Neto, E. P. S. , Custaud, M. A. , Cejka, J. C. , Abry, P. , Frutoso, J. , Gharib, C. , & Flandrin, P. (2004). Assessment of cardiovascular autonomic control by the empirical mode decomposition. Methods of Information in Medicine, 43, 60–65. 15026839

[phy270033-bib-0029] Ng, F. L. , Davis, A. J. , Jepps, T. A. , Harhun, M. I. , Yeung, S. Y. , Wan, A. , Reddy, M. , Melville, D. , Nardi, A. , Khong, T. K. , & Greenwood, I. A. (2011). Expression and function of the K+ channel KCNQ genes in human arteries. British Journal of Pharmacology, 162(1), 42–53. 10.1111/j.1476-5381.2010.01027.x 20840535 PMC3012405

[phy270033-bib-0030] Nunes, F. B. , Graziottin, C. M. , Carlos, J. , Filho, F. A. , Lunardelli, A. , Pires, M. G. S. , Wächter, P. H. , & De Oliveira, J. R. (2003). An assessment of fructose‐1,6‐bisphosphate as an antimicrobial and anti‐inflammatory agent in sepsis. Pharmacological Research, 47, 35–41. 10.1016/s1043-6618(02)00255-4 12526859

[phy270033-bib-0031] Pocivavsek, A. , Baratta, A. M. , Mong, J. A. , & Viechweg, S. S. (2017). Acute Kynurenine Challenge Disrupts Sleep‐Wake Architecture and Impairs Contextual Memory in Adult Rats. Sleep, 40(11), zsx141. 10.1093/sleep/zsx141 29029302 PMC5806560

[phy270033-bib-0032] Porta, A. , Casali, K. R. , Casali, A. G. , Tobaldini, E. , Montano, N. , Lange, S. , Geue, D. , Cysarz, D. , & Van Leeuwen, P. (2021). Temporal asymmetries of short‐term heart period variability are linked to autonomic regulation. American Journal of Physiology—Regulatory, Integrative and Comparative Physiology, 295, R550–R557. 10.1152/ajpregu.00129.2008 18495836

[phy270033-bib-0033] Porta, A. , Montano, N. , Furlan, R. , Cogliati, C. , Guzzetti, S. , Malliani, A. , Chang, H. S. , Staras, K. , & Gilbey, M. P. (2004). Automatic classification of interference patterns in driven event series: Application to single sympathetic neuron discharge forced by mechanical ventilation. Biological Cybernetics, 91, 258–273. 10.1007/s00422-004-0513-3 15378378

[phy270033-bib-0034] Rajendra Acharya, U. , Paul Joseph, K. , Kannathal, N. , Lim, C. M. , & Suri, J. S. (2007). Heart rate variability: A review. Medical and Biological Engineering and Computing, 44(12), 1031–1051. 10.1007/s11517-006-0119-0 17111118

[phy270033-bib-0035] Rigatto, K. , Casali, K. R. , Shenoy, V. , Katovich, M. J. , & Raizada, M. K. (2013). Diminazene aceturate improves autonomic modulation in pulmonary hypertension. European Journal of Pharmacology, 713(1–3), 89–93. 10.1016/j.ejphar.2013.04.017 23665493 PMC3712651

[phy270033-bib-0036] Roig, T. , Bartrons, R. , & Bermtidez, J. (1997). Exogenous fructose 1,6‐bisphosphate reduces K+ permeability in isolated rat hepatocytes, 273(2 Pt 1), C473–C478. 10.1152/ajpcell.1997.273.2.C473 9277344

[phy270033-bib-0037] Sakakibara, K. , Feng, G. , Li, J. , Akahori, T. , Yasuda, Y. , Nakamura, E. , Hatakeyama, N. , Fujiwara, Y. , & Kinoshita, H. (2015). Kynurenine causes vasodilation and hypotension induced by activation of KCNQ‐encoded voltage‐dependent K þ channels. Journal of Pharmacological Sciences, 129(1), 31–37. 10.1016/j.jphs.2015.07.042 26318674

[phy270033-bib-0038] Sakr, Y. , Jaschinski, U. , Wittebole, X. , Szakmany, T. , Lipman, J. , Ñamendys‐silva, S. A. , Martin‐loeches, I. , Leone, M. , Lupu, M. N. , Vincent, J. L. , & ICON Investigators . (2012). Sepsis in Intensive Care Unit Patients: Worldwide Data From the Intensive Care over Nations Audit. Open Forum Infectious Diseases, 5, ofy313. 10.1093/ofid/ofy313 PMC6289022 30555852

[phy270033-bib-0039] Sassi, R. , Cerutti, S. , Lombardi, F. , Malik, M. , Huikuri, H. V. , Peng, C. , Schmidt, G. , & Yamamoto, Y. (2015). Advances in heart rate variability signal analysis : joint position statement by the e‐Cardiology ESC Working Group and the European Heart Rhythm Association co‐endorsed by the Asia Pacific Heart Rhythm Society. Ep Europace, 17, 1341–1353. 10.1093/europace/euv015 26177817

[phy270033-bib-0040] Stone, T. W. , & Darlington, L. G. (2002). Endogenous kynurenines as targets for drug discovery and development. Nature Reviews Drug Discovery, 1(8), 609–620. 10.1038/nrd870 12402501

[phy270033-bib-0041] Stone, T. W. , Stoy, N. , & Darlington, L. G. (2013). An expanding range of targets for kynurenine metabolites of tryptophan. Trends in Pharmacological Sciences, 34(2), 136–143. 10.1016/j.tips.2012.09.006 23123095

[phy270033-bib-0042] Taccone, F. S. , Scolletta, S. , Franchi, F. , Donadello, K. , & Oddo, M. (2013). Brain perfusion in sepsis. Current Vascular Pharmacology, 11, 170–186. 10.2174/1570161111311020007 23506496

[phy270033-bib-0043] Tattevin, P. , Monnier, D. , Tribut, O. , Dulong, J. , Mourcin, F. , Uhel, F. , Le Tulzo, Y. , & Tarte, K. (2010). Enhanced indoleamine 2, 3‐dioxygenase activity in patients with severe sepsis and septic shock. The Journal of Infectious Diseases, 201, 956–966. 10.1086/650996 20151841

[phy270033-bib-0044] Vanderlei, L. C. M. , Pastre, C. M. , Hoshi, R. A. , Carvalho, T. D. , & de Godoy, M. F. (2009). Basics notions of heart rate variability and its clinical applicability. Brazilian Journal of Cardiovascular Surgery, 24(2), 205–217. 10.1590/S0102-76382009000200018 19768301

[phy270033-bib-0045] Wang, Q. , Zhang, M. , Ding, Y. , Wang, Q. , Zhang, W. , Song, P. , & Zou, M. (2013). Activation of NAD(P)H oxidase by tryptophan‐derived 3‐hydroxykynurenine accelerates endothelial apoptosis and dysfunction in vivo. Circulation Research, 114, 480–492. 10.1161/CIRCRESAHA.114.302113 24281189 PMC4104160

[phy270033-bib-0046] Wang, Y. , Liu, H. , Mckenzie, G. , Witting, P. K. , Stasch, J. , Hahn, M. , Changsirivathanathamrong, D. , Wu, B. J. , Ball, H. J. , Thomas, S. R. , Kapoor, V. , Celermajer, D. S. , Mellor, A. L. , Keaney, J. F., Jr. , Hunt, N. H. , & Stocker, R. (2010). Kynurenine is an endothelium‐derived relaxing factor produced during inflammation. Nature Medicine, 16(3), 279–285. 10.1038/nm.2092 PMC3556275 20190767

[phy270033-bib-0047] Wolowczuk, I. , Hennart, B. , Leloire, A. , Bessede, A. , Soichot, M. , Taront, S. , Caiazzo, R. , Raverdy, V. , Pigeyre, M. , ABOS Consortium , Guillemin, G. J. , Allorge, D. , Pattou, F. , Froguel, P. , & Poulain‐Godefroy, O. (2021). Tryptophan metabolism activation by indoleamine 2, 3‐dioxygenase in adipose tissue of obese women: An attempt to maintain immune homeostasis and vascular tone. American Journal of Physiology—Regulatory, Integrative and Comparative Physiology, 303, 135–143. 10.1152/ajpregu.00373.2011 22592557

[phy270033-bib-0048] Worton, S. A. , Pritchard, H. A. T. , Greenwood, S. L. , Alakrawi, M. , Heazell, A. E. P. , Wareing, M. , Greenstein, A. , & Myers, J. E. (2021). Kynurenine relaxes arteries of normotensive women and those with preeclampsia. Circulation Research, 128, 1679–1693. 10.1161/CIRCRESAHA.120.317612 33656370 PMC8154175

[phy270033-bib-0049] Yin, X. , Xin, H. , Mao, S. , Wu, G. , & Guo, L. (2019). The role of autophagy in sepsis protection and injury to organs. Frontiers in Physiology, 10, 1071. 10.3389/fphys.2019.01071 31507440 PMC6716215

