# Supplementary material for: Fructose‐1,6‐bisphosphate reverses hypotensive effect caused by L‐kynurenine in Wistar male rats
Source: Physiol Rep. 2024 Oct 13;12(19):e70033. doi: 10.14814/phy2.70033 (PMC11471349; doi:10.14814/phy2.70033)
Supplement: Supplementary file 1 — Data S1. [file PHY2-12-e70033-s001.docx]

**TITLE PAGE**

**Title:** Fructose-1,6-bisphosphate reverses hypotensive effect caused by L-kynurenine in *Wistar* Rats

**Authors**: Anderson Velasque Catarina^a^; Gisele Branchini^a^; Rafael Andrade Caceres^b^; Renata Streck Fernandes^c^; Bruna Pasqualotto Costa^d^; Kleiton Lima De Godoy Machado^e^; Tiago Becker^f^; Luis Fernando Ferreira^g,h^*; Katya Rigatto^c^; Jarbas Rodrigues de Oliveira^d^; and Fernanda Bordignon Nunes^a,d^

**Affiliation**:

^a^ Graduation Program in Pathology – Laboratory of Computational, Molecular, and Cellular Biophysics - Universidade Federal de Ciências da Saúde de Porto Alegre (UFCSPA), Brazil.

^b^ Department of Diagnostic Methods, Universidade Federal de Ciências da Saúde de Porto Alegre (UFCSPA), Brazil.

^c^ Graduation Program in Health Sciences – Laboratory of Translational Physiology - Universidade Federal de Ciências da Saúde de Porto Alegre (UFCSPA), Brazil.

^d^ Laboratory of Inflammation and Cellular Biophysics - Pontifícia Universidade Católica do Rio Grande do Sul (PUCRS), Brazil.

^e^ Department of Plant Biology, Universidade Federal de Viçosa (UFV), Viçosa, Brazil.

^f^ Department of Mechanical Engineering, Universidade Federal do Rio Grande do Sul, Porto Alegre, Brazil.

^g^ School of Electronics, Electrical Engineering and Computer Sciences, Queen’s University of Belfast, United Kingdom.

^h^ Graduation Program in Medicine: hepatology , Universidade Federal do Rio Grande do Sul, Porto Alegre, Brazil.

**E-mails and ORCID:**

- Anderson Velasque Catarina; e-mail: [andersonvc@ufcspa.edu.br](mailto:andersonvc@ufcspa.edu.br); ORCID: 0000-0003-4148-3857
- Gisele Branchini; e-mail: [giseleb@ufcspa.edu.br](mailto:giseleb@ufcspa.edu.br); ORCID: 0000-0003-2524-8603
- Rafael Andrade Caceres; e-mail: [caceres.bioinformatics@gmail.com](mailto:caceres.bioinformatics@gmail.com); ORCID: 0000-0002-7752-975X
- Renata Streck Fernandes; e-mail: [renatastreck@gmail.com](mailto:renatastreck@gmail.com).
- Bruna Pasqualotto Costa; e-mail: [brupcosta@gmail.com](mailto:brupcosta@gmail.com); ORCID: 0000-0003-4810-4717
- Kleiton Lima de Godoy Machado; e-mail: [kleiton.machado@acad.pucrs.br](mailto:kleiton.machado@acad.pucrs.br); ORCID: 0000-0002-7935-4439
- Tiago Becker; e-mail: [tiago.becker@ufrgs.br](mailto:tiago.becker@ufrgs.br); ORCID: 0000-0001-9762-4466
- Luis Fernando Ferreira; e-mail: [proffernandof@gmail.com](mailto:proffernandof@gmail.com); ORCID: 0000-0002-9496-4884
- Katya Rigatto; e-mail: [kvr@ufcspa.edu.br](mailto:kvr@ufcspa.edu.br); ORCID: 0000-0001-7773-2902
- Jarbas Rodrigues de Oliveira; e-mail: [jarbas@pucrs.br](mailto:jarbas@pucrs.br); ORCID: 0000-0003-0705-1639
- Fernanda Bordignon Nunes; e-mail: [fernandabn@ufcspa.edu.br](mailto:fernandabn@ufcspa.edu.br); ORCID: 0000-0003-1028-7490

**Data availability statement:** The data that support the findings of this study are available from the corresponding author upon reasonable request.

**Funding statement**: This study was financed in part by the Coordenação de Aperfeiçoamento de Pessoal de Nível Superior – Brasil (CAPES) – Finance Code 001

**Conflict of interest disclosure**: The authors declare no conflict of interest.

**Ethics approval statement**: The experimental protocol was approved by the Ethics Committee on the Use of Animals (ECUA) of UFCSPA (protocol number 249/19).

**Patient consent statement**: not applicable.

**Permission to reproduce material from other sources**: not applicable.

****Corresponding Author:***

Luis Fernando Ferreira. School of Electronics, Electrical Engineering and Computer Sciences, Queen’s University of Belfast, 16 Malone Road, Belfast, BT9 6SB, Northern Ireland, United Kingdom. E-mail: [proffernandof@gmail.com](mailto:proffernandof@gmail.com) Phone: 55 51 980707053
